# Shear Wave Elastography and Thyroid Imaging Reporting and Data System (TIRADS) for the Risk Stratification of Thyroid Nodules—Results of a Prospective Study

**DOI:** 10.3390/diagnostics12010109

**Published:** 2022-01-04

**Authors:** Manuela Petersen, Simone A. Schenke, Jonas Firla, Roland S. Croner, Michael C. Kreissl

**Affiliations:** 1 Department of General, Visceral, Vascular and Transplant Surgery, University Hospital Magdeburg, 39120 Magdeburg, Germany; roland.croner@med.ovgu.de; 2 Division of Nuclear Medicine, Department of Radiology and Nuclear Medicine, University Hospital Magdeburg, 39120 Magdeburg, Germany; simoneschenke@web.de (S.A.S.); firla.jonas@gmail.com (J.F.); michael.kreissl@med.ovgu.de (M.C.K.); 3 Department and Institute of Nuclear Medicine, Hospital Bayreuth, 95445 Bayreuth, Germany; 4 Research Campus STIMULATE, Otto-von-Guericke University, 39106 Magdeburg, Germany

**Keywords:** thyroid nodule, shear wave elastography, ultrasound, TIRADS, risk stratification, thyroid cancer

## Abstract

Purpose: To compare the diagnostic performance of thyroid imaging reporting and data system (TIRADS) in combination with shear wave elastography (SWE) for the assessment of thyroid nodules. Methods: A prospective study was conducted with the following inclusion criteria: preoperative B-mode ultrasound (US) including TIRADS classification (Kwak-TIRADS, EU-TIRADS), quantitative SWE and available histological results. Results: Out of 43 patients, 61 thyroid nodules were detected; 10 nodules were found to be thyroid cancer (7 PTC, 1 FTC, 2 HüCC) and 51 were benign. According to Kwak-TIRADS the majority of benign nodules (47 out of 51, 92.2%) were classified in the low-risk- and intermediate-risk class, four nodules were classified as high-risk (7.8%). When using EU-TIRADS, the benign nodules were distributed almost equally across all risk classes, 21 (41.2%) nodules were classified in the low-risk class, 16 (31.4%) in the intermediate-risk class and 14 (27.4%) in the high-risk class. In contrast, most of the malignant nodules (eight out of ten) were classified as high-risk on EU-TIRADS. One carcinoma was classified as low-risk and one as intermediate-risk nodule. For SWE, ROC analysis showed an optimal cutoff of 18.5 kPa to distinguish malignant and benign nodules (sensitivity 80.0%, specificity 49.0%, PPV 23.5% and NPV 92.6%). The addition of elastography resulted in an increase of accuracy from 65.6% to 82.0% when using Kwak-TIRADS and from 49.2% to 72.1% when using EU-TIRADS. Conclusion: Our data demonstrate that the combination of TIRADS and SWE seems to be superior for the risk stratification of thyroid nodules than each method by itself. However, verification of these results in a larger patient population is mandatory.

## 1. Introduction

For a number of years, there has been an intense debate, especially in Germany, whether surgeries for thyroid nodules are unnecessarily performed. Only every 15th nodule that is removed due to a suspected malignancy is indeed malignant [1]. In addition, a thyroidectomy is often performed also for (supposedly benign) nodular goiter [2].

This is why thyroid specialists call for improvements in preoperative diagnostics [3,4].

The majority of thyroid nodules are incidental findings discovered by imaging examinations for reasons unrelated to the thyroid [5]. The goal of a sonographic assessment of thyroid nodules is to distinguish benign nodules that can be managed conservatively from those with suspicious features, which require further workup. Gray-scaled ultrasound is excellent for the detection and characterization of thyroid nodules, but the accuracy for the differentiation between benign and malignant lesions based on single criteria, such as hypoechogenicity, microcalcification and a taller-than-wide shape is low [6,7]. Different research groups developed US-based tools for stratifying the risk of malignancy of thyroid nodules because of a constellation or a number of suspicious ultrasound features, known as the thyroid imaging reporting and data system (TIRADS) [8,9,10,11,12,13,14].

Several studies also demonstrated promising results for the use of sonoelastography combined or not combined with conventional US [7,15,16,17,18,19]. Shear wave elastography is a real-time, non-invasive and reproductible imaging technology, which allows the quantitative assessment of tissue according to its stiffness. The tissue elasticity can be calculated by measuring the propagation of the shear-wave, which is directly related to the Young’s modulus. Thus, a quantitative estimation of thyroid tissue stiffness (in kilopascals, kPa) can be obtained [20,21]. In 2016, guidelines for sonoelastography of the thyroid gland were published by Cosgrove and colleagues [22]. Although there were many studies published in recent years, it should be noted that there is no generally accepted cutoff value yet for the discrimination of benign and malignant nodules [23,24,25]. The recording of the SWE can be influenced by the arterial pulsation, calcification and cystic components in thyroid nodules. Lymphocytic infiltration and fibrosis modify thyroidal structure and may result in a change in thyroidal stiffness [26]. Another study reported that nodules associated with macro-calcifications or eggshell calcifications showing a high false-positive rate for malignancy on SWE [27]. Bhatia et al. reported no difference in SWE indices between calcified and non-calcified lesions [23].

The objective of our study was to evaluate the diagnostic performance of SWE and TIRADS according to Kwak et al. and EU-TIRADS alone and in combination for the assessment of thyroid nodules [12,14].

## 2. Materials and Methods

This prospective study was approved by the local ethics committee of the Magdeburg University Hospital (No. 129/18 RAD 353, date of approval 9 September 2018). A written informed consent was obtained from all patients.

### 2.1. Patients

Between October 2018 and April 2020, consecutive patients who underwent thyroid surgery were, before surgery, evaluated for inclusion into the study. The reasons for performing surgery were multifactorial, i.e., nodular goiter with suspicious nodules on ultrasound or cytology, or a nodular enlargement of the thyroid resulting in cervical symptoms.

Inclusion criteria were an available preoperative B-mode ultrasound, including TIRADS classification (Kwak-TIRADS, EU-TIRADS), performed on-site during ultrasonography, and quantitative SWE as well as available histological results. We did not examine thyroid nodules with macro-calcifications or eggshell calcifications and nodules with a cystic portion of >75%. In case of thyroid nodules with a cystic portion <75%, the measurement was made in the solid areas. If no surgery was performed the patient was excluded from the study.

A total of 53 patients were initially enrolled. As not all patients underwent surgery, the final study group consisted of 43 patients (25 woman and 18 men) with 61 thyroid nodules.

### 2.2. Thyroid US and SWE

The thyroid US and SWE was performed by one examiner (S.A.S) with experience of more than 5 years with both methods. All relevant clinical information was withheld for this examiner in a blind study. A thyroid scintigraphy was not performed routinely on patients. The result of the elastography had no influence on the indication for surgery. The conventional sonography and SWE were conducted using a GE Logiq S9 GE Medical Systems Information Technologies GmbH, Freiburg, Germany. We used a linear probe with a frequency of 6–15 MHz for B-mode ultrasound and a special transducer that generates a low-frequency shear wave in the range of 50 Hz. Using the speed of propagation of this shear or transverse wave, the modulus of elasticity (Young’s modulus) could be determined.

**Figure 1:**
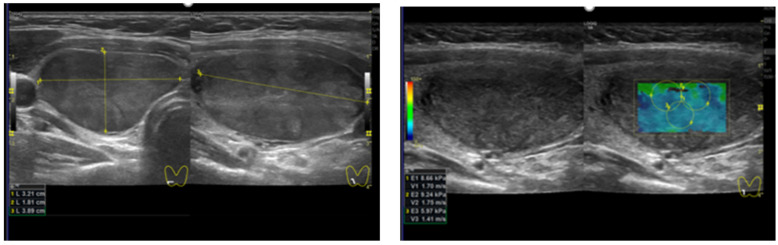
Patient 1 with a TIRADS (Kwak 4B, EU 4) thyroid nodule. The histology final diagnosis was benign. (**left**) B-mode US: solid and hypoechoic; (**right**) quantitative elastosonography showed a value of 7.9 kPa. The yellow lines show the nodule measurement in cm.

**Figure 2:**
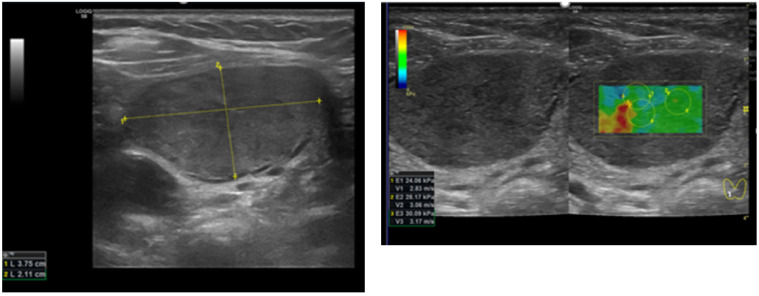
Patient 2 with a TIRADS (Kwak 4B, EU 4) thyroid nodule. The histology final diagnosis was folliculary thyroid carcinoma. (**left**) B-mode US: solid and hypoechoic; (**right**) quantitative elastosonography showed a value of 27.1 kPa. The yellow lines show the nodule measurement in cm.

**Figure 3:**
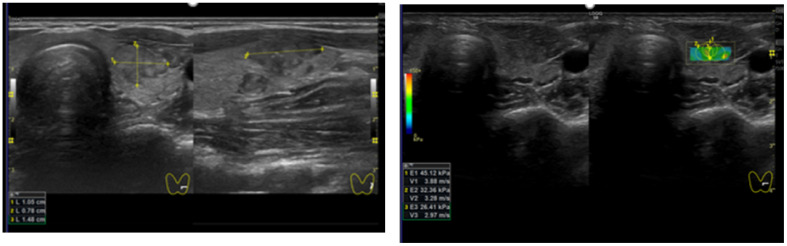
Patient 3 with a TIRADS (Kwak 4C, EU 5) thyroid nodule. The histology final diagnosis was papillary thyroid carcinoma. (**left**) B-mode US: solid, hypoechoic, irregular margin and microcalcification; (**right**) quantitative elastosonography showed a value of 34.6 kPa. The yellow lines show the nodule measurement in cm.

Patients were examined in a supine position with the neck reclined. Three measurements of shear wave propagation were carried out in each case by placing a region of interest (ROI) in the thyroid nodule and in the adjacent thyroid tissue. After that, the mean value of the Young’s modulus was calculated for the nodule and the paranodular normal thyroid tissue (Figure 1, Figure 2 and Figure 3).

Kwak-TIRADS calculates a summed score of suspicious ultrasound characteristics that are present in the thyroid nodules of interest. The number of suspicious ultrasound features (e.g., solid, or almost solid nodule, hypoechogenicity, irregular margins, presence of microcalcifications, and a taller than wide shape, respectively) are used to obtain scores of TIRADS 3, 4A, 4B, 4C or 5 [12].

EU-TIRADS defines four ultrasound features of high suspicion for malignancy (non-oval or round shape, irregular margins, microcalcifications and a marked hypoechogenicity). In contrast to Kwak-TIRADS, EU-TIRADS is divided into low risk, intermediate and high risk [14].

As the number of suspicious features increases, so does the risk for malignancy (Table 1).

In this study, Kwak-TIRADS ≥ 4B and EU-TIRADS ≥ 4 was defined as a cutoff for suspicion of malignancy. The diagnostic performance was tested of either sonoelastography or TIRADS cutoff alone, as well as the combination of TIRADS and SWE.

### 2.3. Statistical Analysis

All statistical tests were performed using IBM SPSS Statistics 24.0 (IBM, Armonk, New York, NY, USA) and WinStat for Microsoft Excel (Version 2012.1.0.96, 2017 R. Fitch Software, Bad Krotzingen, Germany). The results were expressed as mean, standard deviation (SD), median, 25th/75th percentile and range. The variables were tested using the *t*-test, χ^2^-test and the Mann–Whitney test, as indicated. A ROC analysis was performed to calculate the optimal cutoff for Young’s modulus. All results were considered to be significant with *p* < 0.05.

## 3. Results

### 3.1. Patients

Among the 43 patients with a total of 61 thyroid nodules, 25 patients had one nodule and 18 had two nodules. The maximum size of the benign nodules was 21 (13/28) mm and of those with the malignant nodules 17.5 (11.75/35.75) mm, *p* = 0.777.

Histologic examination showed 10 malignant nodules (16%) and 51 benign nodules. We found seven papillary thyroid cancers (PTC), one folliculary thyroid cancer carcinoma (FTC) and two Hürthle cell carcinomas (HüCC).

The mean age of patients with the benign findings were 53.4 ± 13.8 years and of those with the malignant findings 45.5 ± 14.1 years, *p* = 0.18.

The median level of thyroid-stimulating hormone (TSH) in the serum was found to be 2.52 (1.28/3.97) mU/L in the patients with malignant nodules and 0.88 (0.19/1.74) mU/L in the patients with benign findings, *p* = 0.02 (Table 2).

B-mode ultrasound, including TIRADS classification (Kwak-TIRADS, EU-TIRADS).

**Figure 4:**
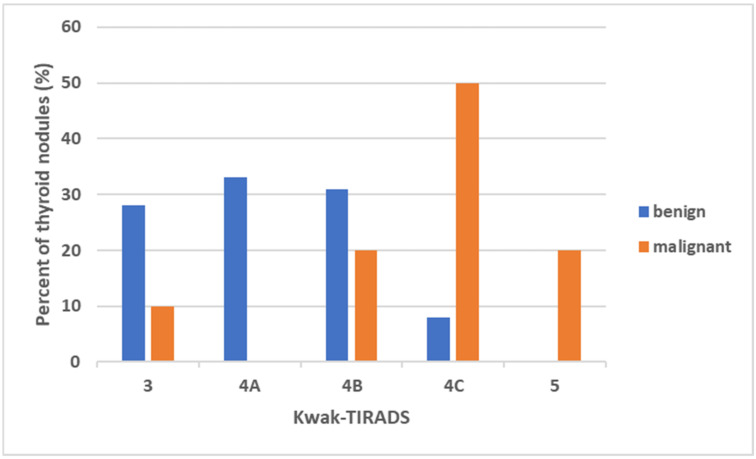
The percentage (y-axis) and the distribution of malignant and benign thyroid nodules according to Kwak-TIRADS. TIRADS = Thyroid Imaging Reporting and Data System.

According to Kwak-TIRADS (Figure 4), seven of ten malignant nodules were classified as high-risk. There were two nodules in the medium-risk class and one nodule in the low-risk class.

The majority of benign nodules (47/51, 92.2%) were classified in the low-risk-and intermediate-risk class. However, four nodules were classified as high-risk (7.8%).

**Figure 5:**
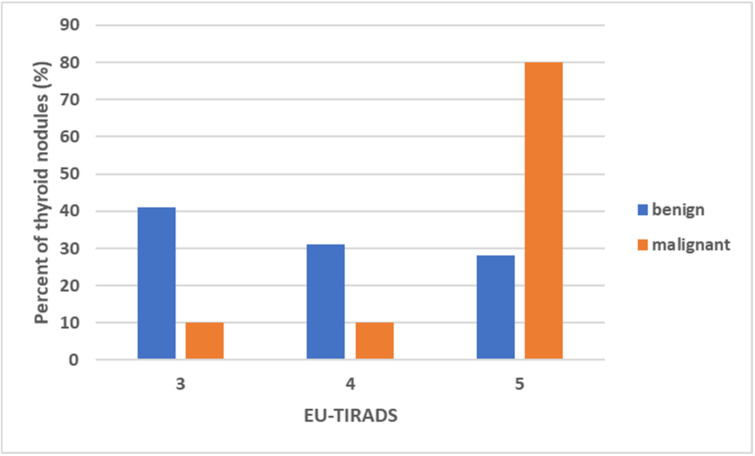
The percentage (y-axis) and the distribution of malignant and benign thyroid nodules according to EU-TIRADS. TIRADS = Thyroid Imaging Reporting and Data System.

The percentage and distribution of malignant and benign thyroid nodules according to EU-TIRADS is presented in Figure 5. The benign nodules showed a wide distribution across all risk classes; 21 (41.2%) nodules were classified as low-risk and 16 (31.4%) as intermediate-risk. Of note, 14/51 (27.4%) of the benign nodules were considered to be high-risk.

In contrast, most of the malignant nodules (eight out of ten) were classified as high-risk. One nodule was classified as low-risk and one as intermediate-risk.

### 3.2. Elastography

The Young’s modulus showed a trend to be higher in malignant thyroid nodules (median 27.6; 25th/75th percentile 17.3/63.4) than in benign nodules (median 19.5; 25th/75th percentile 11.7/32.3) (*p* = 0.22).

In terms of accuracy (ACC), the combination of TIRADS with elastography showed an increase from 65.6 to 82.0% (Kwak-TIRADS) and from 49.2 to 72.1% (EU-TIRADS) (Table 3).

**Figure 6:**
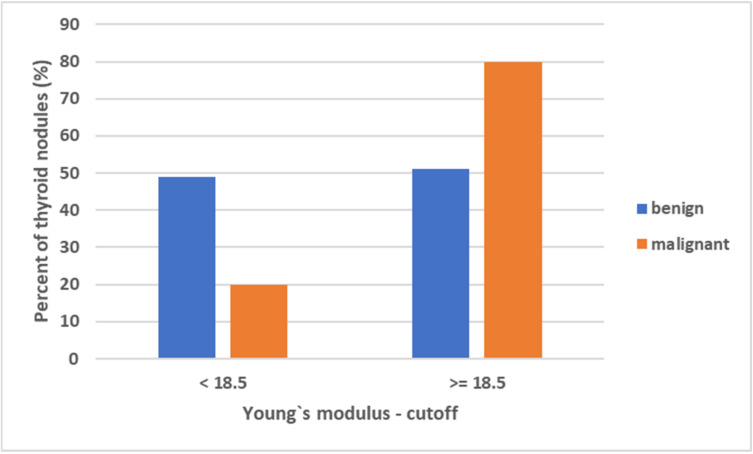
Results of SWE for the optimal cutoff to distinguish malignant from benign nodules calculated by the ROC analysis.

Figure 6 showed the results of SWE for the optimal cutoff (≥18.5 kPa) to distinguish malignant from benign nodules calculated by the ROC analysis (AUC = 0.79, 95% Confidence Interval: 0.61; 0.98).

## 4. Discussion

Suspicious thyroid nodules have specific ultrasonographic features, such as hypoechogenicity, irregular margins, microcalcifications, a solid composition and a taller-than-wide shape [12,13,15,18]. US-based tools for stratifying the malignancy risk of thyroid nodules, using a variety of different ultrasound features (EU-TIRADS, Kwak-TIRADS), help to enhance the inter-observer agreement of descriptions and simplify the communication of results [12,14]. However, the accuracy of US for the differentiation between malignant and benign thyroid nodules remains unsatisfactory [7,28]. The introducing of sonoelastography in the clinical workup of thyroid nodules shows promising results for the differentiation of malignant and benign nodules [24,29].

In our study, we analyzed the diagnostic performance of SWE and grey-scale US using TIRADS alone, and both methods combined, respectively, with histological results as the gold standard. Our results showed a better overall accuracy for the combination of TIRADS and elastography in nodules of intermediate or high risk on TIRADS. We used sonography and elastography to differentiate between benign and malignant nodules without further differentiation. Validating the approach in the various subentities of thyroid cancer lies within the scope of a planned larger trial with higher patient number and a multicentric setting. Specifically, minimally invasive follicular thyroid cancer of course is a great challenge for all involved disciplines and will be difficult to diagnose in an ultrasound-based diagnostic approach. This could also hold true for Huerthle cell carcinoma.

To date, few studies have reported the diagnostic performance combining Kwak-TIRADS or EU-TIRADS with SWE. In addition to predominantly retrospective studies, there are only two prospective Italian studies [30,31]. Comparable to our study, Cantisani et al. also evaluated the diagnostic performance of SWE, SRE alone and in combination with Kwak-TIRADS in 243 nodules [30]. TIRADS alone showed a sensitivity of 59.6%, a specificity of 83.8%, a PPV of 50.0% and an NPV of 88.4% in the overall assessment. SWE (kPa) had a sensitivity and specificity of 67.3% and 82.7%, respectively, with an AUC of 0.75, a PPV of 51.5% and an NPV of 90.3%. The combination of TIRADS with SRE led to a significant increase in the accuracy of the former, although the accuracy of SRE as a standalone method remained higher. Moreover, the addition of SWE and SRE to TIRADS did not allow an increase in the area under the curve in ROC analysis compared to SRE alone. In comparison, our results show a higher sensitivity and a lower specificity for both TIRADS and SWE.

Contrary to our study, Celleti et al. performed an analysis to assess SRE and SWE accuracy alone and with TIRADS classification for the risk stratification of thyroid nodules [31]. They examined the diagnostic performance in a selective patient collective and include only patients with indeterminate results in cytology. Due to this selectivity, there may be a higher specificity and PPV in comparison to our consecutive patient group. The NPV was comparable, so it can be said with a high degree of certainty that a soft thyroid nodule is benign.

Most reports assessed the combination of other TIRADS (ACR-TI-RADS, ATA, K-TIRADS) and SWE.

The literature shows that both TIRADS and SWE could be effectively used to differentiate between benign and malignant thyroid nodules. Some researchers combined Kwak-TIRADS and SWE to assess thyroid nodules and found that SWE could improve the diagnostic performance in category 4 nodules (intermediate risk nodules) [32,33]. Other studies showed that Kwak-TIRADS combined with SWE significantly improved the diagnostic performance for thyroid cancer detection [32,33,34]. Another group of researchers documented in a collective of 187 patients that the specificity significantly rose from 20.1% to 47.0% (*p* < 0.001) without a loss of sensitivity (98.4% for Kwak-TIRADS versus 98.4% for combined Kwak-TIRADS and SWE) [35]. Our data also demonstrated a high sensitivity both for TIRADS alone and in combination with SWE, and it had a similar increase in specificity. Huang et al. reported, in a retrospective approach of 69 patients, similar results to our study [36]. The sensitivity and specificity in correctly diagnosing thyroid nodules were found to be 70.8% and 65.2% for Kwak-TIRADS, 68.8% and 91.3% for SWE, 77.1% and 78.4% for contrast-enhanced ultrasonography, and 91.7% and 95.7% for Kwak-TIRADS + SWE + CEUS. The AUC for Kwak-TIRADS, SWE and CEUS, and Kwak-TIRADS + SWE + CEUS in diagnosing thyroid nodules were 0.68, 0.84, 0.79, and 0.94, respectively. A significant difference was observed between a combination of the three methods as compared to the single use of them (*p* < 0.05) and concluded that combining SWE and CEUS improves the differential diagnosis of Kwak-TIRADS category 4A and 4B nodules [36].

Comparably, we found the AUC = 0.79 (0.61; 0.98) for the optimal cutoff of SWE to distinguish malignant from benign nodules. Zhang et al. described, in combination with SWE, that the sensitivity of Kwak-TIRADS for the diagnosis of nodules was improved, the difference was statistically significant (*p* ≤ 0.001) and the specificity was decreased, but the difference was not statistically significant (*p* > 0.05) [37]. The difference in the AUC was not significant (*p* > 0.05). They concluded, in keeping with our findings, that the combination of TIRADS and SWE had a high performance in the diagnosis of thyroid nodules. Wang et al. found that the elasticity indices were significantly higher in malignant versus benign nodules (*p* ≤ 0.001) [38]. The minimum elasticity index (cutoff, 40.7 kPa) of the stiffest part combined with conventional US showed the highest AUC (0.774) but was not superior to conventional US (0.791). Combined with the standard deviation of the elasticity index for the whole lesion (cutoff, 6.8 kPa), US yielded the highest sensitivity (95.5%; *p* < 0.001) and lowest specificity (42.1%; *p* < 0.001). Sensitivity increased and specificity decreased by adding any other SWE elasticity index. Contrary to our results, the study group concluded that adding SWE to conventional US did not improve diagnostic performance. Similarly, Yoon et al. found that ultrasound was superior to additional imaging modality, such as SWE, with respect to differentiating thyroid nodules [39].

### Limitations

Our study has some limitations. Firstly, only 43 patients were investigated in the prospective design thus far. A verification on a larger group is necessary. A multicenter approach would be useful to collect more data on a larger scale. However, it is known that especially ultrasound assessment but also shear wave elastography is subject to heterogeneity introduced by the equipment used, and it is also observer dependent. Therefore, intense cross-validation is to be performed in order to obtain reliable and valid data. Secondly, the assignment of the nodules to the TIRADS classes and the classification of sonoelastography were performed by only one examiner. Therefore, interobserver variability was not tested in this study. However, several studies have been published concerning the reproducibility of SWE, showing substantial and perfect agreement. With regard to TIRADS classification, the interobserver agreement and diagnostic accuracy were very similar for Kwak-TIRADS and EU-TIRADS [40,41]. Finally, hyperfunctioning thyroid nodules are considered to be benign. Elastography cannot differentiate scintigraphically hyperfunctional from hypofunctional thyroid nodules. Its accuracy in the assessment of at least “hot” thyroid nodules is to be questioned [42]. Schenke et al. investigated how TIRADS classifies hyperfunctioning thyroid nodules. They demonstrated that many hyperfunctional nodules have suspicious features when assessed by TIRADS and that thyroid scintigraphy is essential to prevent unnecessary fine-needle biopsies and thyroid surgeries [43].

## 5. Conclusions

Our data indicate that the combination of TIRADS and SWE seem to be superior for the risk stratification of thyroid nodules at intermediate and high risk than each method alone. However, verification on a larger group of patients is necessary. A multicenter approach is useful to collect more data on a larger scale.

## Figures and Tables

**Table 1:** Comparison between Kwak-TIRADS [12] and EU-TIRADS [14].

	TIRADS 2	TIRADS 3	TIRADS 4			TIRADS 5
Kwak	0%	1.7%	A: 3.3%	B: 9.2%	C: 44.4–72.4%	87%
EU	0%	2–4%	6–17%			26–87%

**Table 2:** Patient characteristics, histological results, maximum diameter and TSH values.

	Malignant Nodules (n = 10)	Benign Nodules (n = 51)
Age (y) Mean ± standard deviation	45.5 ± 14.1	53.4 ± 13.8
Gender Female/male	6/2	19/16
Histology		
Papillary thyroid cancer (PTC)	7	
Folliculary thyroid cancer (FTC)	1	
Hürthle cell carcinoma (HüCC)	2	
Maximum size (mm) Median (25th/75th percentile)	17.5 (11.8/35.8)	21 (13/28)
TSH (mU/L) Median (25th/75th percentile)	2.52 (1.28/3.97)	0.88 (0.19/1.74)

**Table 3:** TIRADS and SWE alone and TIRADS combined with SWE: PPV, NPV, sensitivity, specificity and ACC.

	PPV (%)	NPV (%)	Sensitivity (%)	Specificity (%)	ACC (%)
Kwak-TIRADS ≥ 4B	31.0	96.9	90.0	60.8	65.6
EU-TIRADS ≥ 4	23.1	95.5	90.0	41.2	49.2
Young’s modulus ≥ 18.5 kPa	23.5	92.6	80.0	49.0	54.1
Kwak-TIRADS ≥ 4B + Young’s modulus	47.1	95.5	80.0	82.4	82.0
EU-TIRADS ≥ 4 + Young’s modulus	34.8	94.7	80.0	70.6	72.1

Abbreviations: PPV = positive predictive value; NPV = negative predictive value; ACC = accuracy; TIRADS = thyroid imaging reporting and data System; SWE = shear wave elastography.

## Data Availability

The data that support the findings of this study are available from the corresponding author upon reasonable request.
